# Factor VII Gene Defects: Review of Functional Studies and Their Clinical Implications

**DOI:** 10.29252/.23.3.165

**Published:** 2019-05

**Authors:** Shirin Shahbazi, Reza Mahdian

**Affiliations:** 1 Department of Medical Genetics, Faculty of Medical Sciences, Tarbiat Modares University, Tehran, Iran; 2 Molecular Medicine Department, Pasteur Institute of Iran, Tehran, Iran

**Keywords:** Factor VII deficiency, *in vitro* techniques, mutation

## Abstract

Coagulation factors belong to a family of plasma glycosylated proteins that should be activated for appropriate blood coagulation. Congenital deficiencies of these factors cause inheritable hemorrhagic diseases. Factor VII (FVII) deficiency is a rare bleeding disorder with variable clinical symptoms. Various mutations have been identified throughout the *F7* gene and can affect all the protein domains. The results of previous experiments have partly revealed the correlation between genotype and phenotype in patients with FVII deficiency. Nevertheless, each particular variant may affect the coagulative function of FVII, mainly via altering its expression level, extra-cellular secretion, tissue factor binding affinity, or proteolytic activity. The pathogenicity of the variants and molecular mechanisms responsible for clinical symptoms in patients with FVII deficiency should be characterized via *in silico* and *in vitro*, as well as *in vivo* functional studies. This review has highlighted the most important functional studies reported on *F7* gene variants, including relevant reports regarding Iranian FVII deficiency patients.

## INTRODUCTION

### Thrombosis and coagulation factors

**Fig. 1:**
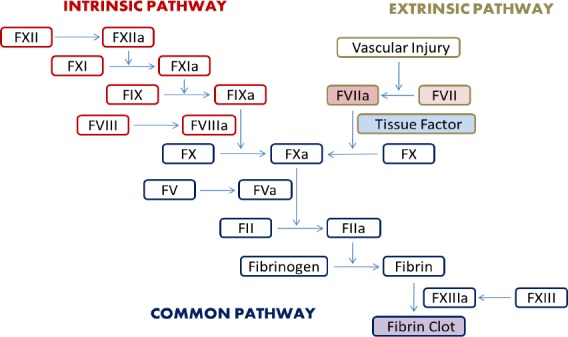
Schematic display of the coagulation cascade. Coagulation factors are mainly enzymes with protease catalytic activities. Upon activation of initial coagulation factors such as FXII (intrinsic pathway) or FVII (extrinsic pathway), consecutive processes are triggered, which ultimately convert fibrinogen to fibrin clot and maintain hemostasis. Tissue factor plays a pivotal role in the extrinsic pathway via converting FVII to its active form FVIIa.

In the coagulation process, which ultimately prevents bleeding, various mechanisms such as vascular contraction, platelet aggregation, and clot formation are activated[1,2]. The coagulation cascade (Fig. 1) is triggered by the tearing of vessel and progress through complex sets of biochemical reactions that are carried out by blood coagulation factors[1-3]. Coagulation factors belong to a family of plasma glycosylated proteins that should be activated for appropriate blood coagulation[4]. In general, coagulation factors are present in plasma at very low levels and are dependent on vitamin K for their activity[5]. Congenital deficiencies of these factors cause inheritable hemorrhagic diseases, which are often rare[6]. Defective function of coagulation factors can be quantitative or qualitative. In qualitative type, although functional tests may indicate coagulation factor deficiency, antigen detection assays show that their plasma level is normal or increased[7]. The main consequence of coagulation cascade is the formation of active substances that are called prothrombin activators[8]. The prothrombin activators catalyze the conversion of prothrombin to thrombin, which converts fibrinogen into fibrin fibers. Eventually, fibrin fibers trap the platelets and form the clot. The prothrombin activators are formed in two ways that interact with each other: (1) the extrinsic pathway that starts with damage to the vascular walls and their surrounding tissues and (2) the intrinsic pathway that starts inside the blood. Factor VII (FVII) plays a pivotal role in the commencement of blood coagulation through the extrinsic pathway[9].

### Coagulation factor VII

FVII is a serine protease produced in the liver and presents in plasma as a zymogen at a concentration of 10 nM (0.5 µg/ml)[10-12]. This vitamin K-dependent glycoprotein is circulating in plasma in two forms, mainly as inactive single-chain zymogen and partly as active form (FVIIa) consisted of heavy and light chains. Following vascular injury, FVII is converted into its active form and binds to the tissue factor (TF) to form the TF/FVIIa complex. The conversion of FVII to FVIIa occurs by breaking the peptide linkage between amino acids Ile153 and Arg152[13]. TF consists of phospholipids derived from tissue membranes plus lipoprotein complexes of damaged tissue. The TF/FVIIa complex acts as an enzyme on Factor X (FX) and converts it into its active form (FXa) in the presence of calcium ion. FXa is rapidly combined with tissue phospholipids, a part of TF, or released from the platelets[14]. Together with FV, they form the prothrombin-activating complex. Then this complex converts prothrombin into thrombin in the presence of calcium ion, and the coagulation process proceeds[3,15].

### Factor VII biosynthesis and functions

The coagulation factors (FVII, FIX, and FX) and prothrombin have almost the same protein structure characteristics. All of these proteins have a signal peptide that is necessary for their transmission to the endoplasmic reticulum. They also contain a pro-peptide sequence that carries out vitamin K-dependent γ-carboxylation in mature protein and is cleaved after transferring to Golgi’s system[16,17]. The FVII protein also contains two epidermal growth factor-like (EGF-like) domains and an activation peptide with a glycosylated asparagine that provides a proteolytic cleavage site. The catalytic regions exhibit the serine protease activity, which leads to various functions of the protein[18]. The role of FVII in the pathogenesis of various cancers has extensively been studied[19-24]. Though the molecular pathogenesis of the increased expression of FVII by cancer cells has not precisely been described, the ectopic expression of FVII may promote the division, migration, and invasion of cancer cells. This process seems to be mainly mediated through the TF/FVIIa/PAR2 complex[25]. Recently, it has been shown that FVII is an important target of androgen receptor in breast cancer cells. That study indicated that the androgen receptor binds to the *F7* promoter near the ATG translation start codon, which suggests that the androgen receptor directly activates *F7* gene expression in cancer cells[19].

### Factor VII deficiency

The deficiency of FVII was first described in 1951. The disease is known as a hereditary bleeding disorder with prevalence of 1 in every 300,000- 500,000 individuals[7,26]. Children with congenital FVII deficiency may be diagnosed following a gastrointestinal tract or central nervous system bleeding in the first six months of their life[27,28]. Patients with severe FVII deficiency may experience joint and muscle bleeding, easy bruising, and post-operative hemorrhage. Bleeding can also occur spontaneously in the mouth, the nose, the genitals, and urinary tract[26]. Furthermore, the affected women often suffer from severe menorrhagia[29]. In sum, FVII deficiency is a rare bleeding disorder with variable clinical symptoms[28-37]. However, in many cases, there is no direct correlation between the factor plasma levels and the severity of the disease symptoms[28-30,32,38]. In fact, some people with very low levels of FVII may demonstrate mild symptoms. In cases of very low factor levels, the clinical manifestation of the disease may be similar to hemophilia symptoms. However, the patients are generally treated with the administration of recombinant FVII[33,39,40].

### Factor VII gene (*F7*)

**Fig. 2:**
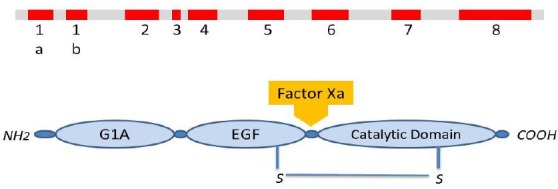
Schematic illustration of factor VII gene (*F7*) and its encoded protein (FVII). Upper: *F7* gene contains nine exons that encode different parts of the protein. Exons 1a and 1b, pre-propeptide; exons 2 and 3, G1a domain; exon 4, EGF-1 domain; exon 5, EGF-2 domain; exons 6 and 7, activation region; exon 8, serine protease catalytic domain. Lower: Mature FVII is a 50-kDa protein of 406 amino acids. Upon contact with tissue factor exposed by vascular injury, FVII is cleaved into its two-chain active form (FVIIa), mainly by factor Xa. The light chain of FVIIa comprises a Gla domain and two EGF domains, whereas the heavy chain contains the serine protease catalytic domain, which is structurally homologous to those of the other coagulation factors[63,71].

**Fig. 3:**
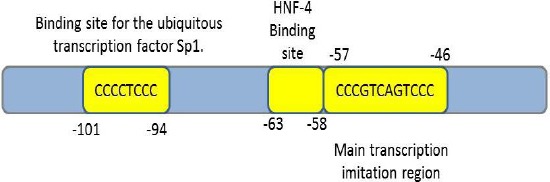
Structure of *F7* gene promoter region. The region spanning [-58 to -63] bases on *F7* promoter has been assigned as HNF-4 binding site, while the [-94 to -101] region provides the binding site for transcription factor Sp1. The -94C>G and the -61T>G homozygous promoter mutations are known to cause severe FVII deficiency by impairing the binding of the Sp1 and HNF-4 transcription factors, respectively[44].

The *F7* gene is located on chromosome 13 (13q34). This gene has nine exons and eight introns, which, besides the gene promoter region, composes a 12-kb gene locus near the telomeric region of the chromosome[41]. Two other genes (i.e. *FX* and *PROZ*) which encode vitamin K-associated proteins, are also located close to the *F7* gene locus. The complete sequence of this gene was reported in 1987 by O’Hara *et al*.[42]. The length of the introns in this gene varies between 68 nucleotides and 2.6 kb, while the gene exons are between 25 nucleotides and 1.6 kb. The exons 1a, 1b, and a part of the exon 2 join together and encode the pre-pro leader sequence. The presence or absence of exon 1b assigns the pre-pro leader with a size of 60 or 38 amino acids, respectively. Both variants are naturally occurring in humans, although the lack of exon 1b is more common[43]. The rest of the exon 2 plus the remaining exons encode the mature protein. Regardless of the transcribed alternate exons, the mature FVII protein in the plasma is a single chain protein with a molecular weight of 50 kDa that contains 406 amino acids. In contrast, FVIIa has a light chain (gamma carboxy-glutamic acid domain and two other EGF-like domains) and a heavy chain with catalytic activity (Fig. 2). The promoter and regulatory regions of this gene have extensively been studied[44-49]. The main transcription initiation region is located at [-57CCCGTCAGTCCC-46] upstream of the transcription starting point. The binding region for the transcription factor HNF4, which affects the expression of other genes in the liver, spans bases from -63 to -58 (Fig. 3). There is also a gene locus on chromosome 8 that may play a role in regulating FVII levels. The presence of this locus was suggested by observing FVII deficiency in patients with trisomy of chromosome 8. Eventually, Fagan *et al*.[50] have assigned this regulatory locus on 8p23.2-p23.1 chromosomal region.

### *F7* gene mutations

**Fig. 4:**
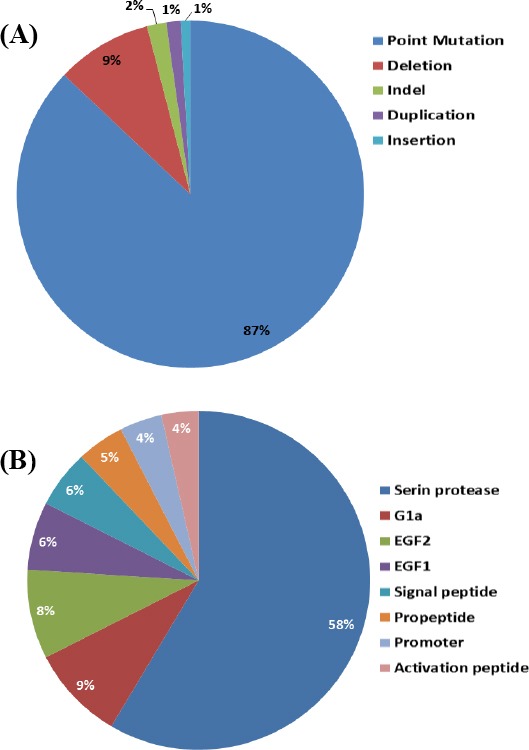
(A) The mutational spectrum of *F7* gene including different types of variants. Point mutations and gene deletions comprise more than 90% of these variants. Interestingly, most of the point mutations in coding sequence of the gene are missense. (B) Domains of the FVII protein affected by the variants in the corresponding *F7* gene regions. Exon 8, which encodes the largest domain of the protein (i.e. serine protease catalytic domain), harbors most of the gene variants identified so far.

According to the reported data (http://www.factorvii.org), the *F7* gene harbors more than 200 different variants. These variants include missense, nonsense, small insertion/deletion, and splice site mutations, which may affect every region of the gene (Fig. 4A), and the mutations identified throughout the *F7* gene can affect all the protein domains (Fig. 4B). Although *F7* mutations are very heterogeneous, some are common in particular populations[51]. On the other hand, many patients with a specific mutation in the *F7* gene may have no significant clinical manifestation. Currently, comprehensive information regarding these mutations is available at the FVII gene variants database (http://www.factorvii.org)[26]. Point mutations are the main causes of FVII inherited defects, where missense mutations are the most frequent variants. Exon 8 is the largest exon of the gene and harbors a large number of mutations. Like other hereditary coagulation defects, such as FIX deficiency (hemophilia B), many mutations occur in CpG hot spot regions. To date, frequent examples of such mutations have been described (R79Q/W, 6071G>A, A244V, R304Q, and T359M)[52]. In a comprehensive study on 717 patients in Latin America and Europe, 131 mutations were observed in 73 homozygotes, 145 heterozygote compounds, and 499 heterozygotes patients, of which 71% of homozygous and 50% of compound heterozygotes cases were symptomatic. Interestingly, despite the observation of FVII deficiency symptoms in some patients, almost 10% of the patients had no mutations in the screening analysis[30]. Whether the FVII deficiency is due to the defects in genes other than *F7* has yet to be described. It is also believed that the plasma levels of the factor are regulated by *F7* gene polymorphisms. However, their effect on the severity of patients’ clinical manifestation is not clear. In general, the most severe cases are either homozygous or compound heterozygous with FVII: C levels less than 2.0% of normal, but occasionally, heterozygous carriers display hemorrhagic symptoms that can be severe in rare cases. For instance, a heterozygous 19-year-old patient with severe spontaneous intracranial bleeding was reported; the patient had no previously recorded hemorrhagic symptoms[53].

### Functional studies on *F7* gene variants

Up to now, various functional studies[48,54,55] have been conducted to show the effects of *F7* gene variants, mainly on the secretion rate, ligand binding, and coagulation activity of the protein (Table 1). These studies are often based on the *in vitro* expression of mutant recombinant FVII in mammalian cells in culture. Their results partly revealed the correlation between the genotype and phenotype in the patients with FVII deficiency. Nevertheless, each particular variant may affect the coagulative function of FVII, chiefly via altering its expression level, extracellular secretion, TF-binding affinity, or proteolytic activity. Functional analysis of the *F7* mutations has demonstrated that the binding of FVII to TF occurs through a large interface between the two proteins, which comprise all four FVIIa domains and two TF extracellular domains[54,56]. The Gla domain binds to the C-terminal membrane domain, while EGF1 interacts with both domains of TF. EGF2 and the FVII protease domains form a merged surface interacting with the N-terminal of the TF. Although the mechanism by which TF increases the catalytic activity of FVIIa is not well known, previous studies have indicated that different *F7* gene variations can change this interaction and decrease the coagulation activity of the protein[54]. For instance, the R79Q mutation has no effect on the expression of the FVII protein but decreases its TF binding affinity[57,58]. Protein structure analysis by X-ray crystallography has displayed that the region that contains this residue plays an important role in the interaction of EGF1 with TF[54]. In the same way, the Q100R mutation may affect the protein expression and cause defective FVIIa/TF complex formation[59]. Peyvandi *et al*.[60] have studied Pro303Thr variant in an Iranian patient with relatively severe hemorrhage. The functional study of this mutation was performed using *in vitro* expression of the defective FVII protein, followed by biochemical coagulation tests. The mutation was induced by site-directed mutagenesis in exon 8 of *F7* gene, and the mutated protein was expressed in mammalian cells. Quantitative tests have suggested that the expression and the secretion of the mutated protein were normal. However, further experiments have revealed that impaired binding of FVII to the TF diminishes its proteolytic activity[60]. It has been reported that mutant FVII protein with R152Q mutation has no detectable activity. This mutation occurs at the proteolytic cleavage site required for the conversion of FVII into FVIIa. Thus, the mutation affects the protein activity by inhibiting the activation of FVII serine proteases[58]. In another functional analysis, although the F328S variant led to partially diminished TF binding, the protein was not able to activate FX, possibly due to a defective substrate binding site[61].

**Table 1:** Summary of the most important functional studies on the FVII molecular defects subsequent to *F7* gene variants detected in FVII deficiency patients

Gene/protein region	Variant	Clinical pathogenicity	Functional defect	Method	Ref.
SPCD	G420V	PA	LS	ELISA/CM	[55]
SPCD	p.I289del	PA	LS	ELISA/CM	[55]
SPCD	A354V-p.P464Hfs	LP	LS	ELISA/CM	[55]
SPCD	H348R	PA	LS	ELISA/FM	[67]
SPCD	S282R	PA	LS	ELISA/FM	[67]
EGF-like-2 domain	C91S	PA	LCA	IHC/ELISA/CA	[62]
SPCD	Cys329Gly	PA	LCA	ELISA/CA	[63,65]
EGF-like-1 domain	R79Q	PA	LTB	ELISA/CA	[57,58]
SPCD	R152Q	PA	LCA	ELISA/CA	[58]
Promoter	-2989C/A	LP	HE	FACS	[48]
Promoter	-670A/C	PA	LE	FACS	[48]
Promoter	-630A/G	PA	HE	FACS	[48]
Promoter	-402G/A	PA	HE	FACS	[48]
Promoter	-401G/T	LP	HE	FACS	[48]
Promoter	-323ins0/10	LP	LE	FACS	[48]
Promoter	-122T/C	LP	LE	FACS	[48]
Intronic (IVS6)	IVS6 + 1G>T	PA	LE	Western blot/ELISA	[72]
3’ UTR	g.11293_11294insAA	Conditional pathogenicity	Low mRNA expression	ELISA/CA/qRT-PCR	[73]
SPCD	Arg277Cys	LP	Low secretion/ moderate activity	ELISA/CA/qRT-PCR	[73]
SPCD	Arg353Gln	Benign	None	ELISA/CA/qRT-PCR	[73]
Gla domain	Ser23Pro	PA	LTB	Crystallography/CA	[54]
EGF-like-2 domain	Cys135Arg	PA	Disrupted disulfide bond	Crystallography/CA	[54,74]
SPCD	Arg247Cys	PA	LTB	Crystallography/CA	[54]
SPCD	Ser282Arg	PA	LTB	Crystallography/CA	[54]
SPCD	Ser363Ile	PA	LTB	Crystallography/CA	[54]
SPCD	Trp364Cys	PA	LTB	Crystallography/CA	[54]
SPCD	Trp364Phe	PA	LTB	Crystallography/CA	[54]
SPCD	Pro303Thr	PA	LTB	Crystallography/CA/ELISA/ solid-phase binding assay	[54,60]
Gla domain	Phe24del	PA	LTB	Crystallography/CA	[56]
EGF-like-2 domain	Arg110Cys	PA	IPF	Clotting assay/EIA	[18]
EGF-like-2 domain	Asp123Tyr	PA	IPF	Clotting assay/EIA	[18]
Promoter	-94C>G	PA	Low Sp1 binding	Reporter gene expression assay/ electrophoretic mobility shift assay	[75]

SPCD, serine protease catalytic domain; PA, pathogenic; LP, likely pathogenic; LS, low secretion; LCA, low coagulative activity; LTB, low TF binding; IPF, impaired protein folding; HE, high expression; LE, low expression; CM, confocal microscopy; FM, fluorescence microscopy; CA, coagulation assay; Ref. reference

Recently, we have reported the FVII functional defects consequent to C91S mutation in a homozygote patient with mild bleeding symptoms[62]. We expressed the mutant protein in CHO-K1 cells *in vitro* and assessed its properties using coagulation assays and immunocytochemistry. In spite of increased secretion of FVII in the culture medium of the cells expressing the mutant FVII, C91S substitution severely affected the coagulant activity of FVII. The C91S substitution was first reported in a British patient with FVII deficiency[63]. The mutation occurs in the exon 5 of *F7* gene and alters residue 91 in EGF2 (EGF-like 2) domain of the protein. The EGF-like and the serine protease domains are necessary for FVII and TF interaction[64]. Previous studies have also shown that EGF2 mutations dramatically impair FVII coagulant activity by affecting protein-protein interactions[18,54]. The review by Peyvandi *et al*.[54], which included 21 families with FVII deficiency, has identified nine new missense mutations in the Gla, EGF-2, or serine protease domains (Table 1). They analyzed the protein crystal structure to describe the functional effects of these variants on FVIIa and FVIIa/TF complex. In a similar study, Millar *et al*.[63] have evaluated 23 new mutations in 38 British patients with FVII deficiency. They also used crystal structure analysis and molecular modeling of the FVIIa/TF complex to determine the variants pathogenicity. In a study on Italian patients, D’Andrea *et al*.[18] have reported a 6-year-old female with FVII deficiency who was identified as compound heterozygote for Asp123Tyr and Arg110Cys mutations, both of which in the EGF-2 domain. In order to evaluate the importance of the EGF-2 motif and the pathogenicity status of the variants, a functional study was performed on the both mutations. When the recombinant variants were expressed in mammalian cells, FVII:C and FVII:Ag were assessed in the cell lysate and culture medium of the host cells. They observed that these mutations decreased the intracellular accumulation and the secretion rate of FVII protein. They concluded that the mutations in EGF-2 domain could affect FVII processing, stability, or secretion[18]. Also, the effect of Gly97Cys and Gln100Arg mutations on FVII secretion and function was studied. These mutations that occur in EGF-2 may alter the intracellular localization and the secretion of the protein. To evaluate the pathogenic outcome of these variants, COS-1 and CHO cells were transfected with expression vectors containing wild type and mutated alleles. The host cells were examined by immunostaining to reveal intracellular localization of FVII protein. The results showed that the mutations in EGF-2 domain can alter the localization pattern as well as the secretion rate of FVII protein[18].

Cysteine residues play an important role in FVII function, in particular, Cys329 that is strongly preserved in the serine proteases is critical for TF binding and, thus, the catalytic function of FVIIa. Disruption of disulfide bond between Cys329 and Cys310 dramatically affects the structure and the function of the protein[65]. So far, numerous patients with Cys329Gly mutation and a patient with Cys329Arg have been reported[63,66]. The molecular mechanisms involved in the pathogenesis of FVII deficiency consequent to the mutations in the serine protease catalytic domain have widely been studied *in vitro*. In a study by Chollet *et al*.[55], CHO-K1 cells were transiently transfected to describe the mechanisms by which these three different mutations reduce the levels of FVII. They revealed impaired secretion of the defective FVII protein in the culture medium. These results were consistent to the low FVII levels measured in patients carrying these mutations. In another study, we performed a functional study on H348R and S282R mutations detected in compound heterozygous status in a FVII-deficient patients[43,67]. The both variants could lead to lowered secretion of the mutant proteins and undetectable coagulation activity *in vitro*.

The expression of chimeric FVII/GFP proteins has been analyzed to identify the effects of nonsense mutations on the biosynthesis and secretion of FVII. Further studies have been conducted to investigate the expression features of *F7* promoter variants. The mutations in the promoter consensus sequence of *F7* gene (-94C> T; -61T> G; -55C> T) affect the binding of transcription factors that are important for the expression of FVII. These three mutations have been studied with the help of reporter genes in transfected cells. The transcripts containing the reporter gene along with the mutated upstream sequences of the *F7* gene showed decreased expression rate compared to the wild-type gene. It has also been shown that 94C>T mutation occurs at SP1 binding site and -61T>G mutation at HNF4 binding sequence. The mutation at -55C>T also caused a significant reduction in the binding affinity of HNF4 to this sequence. The severe clinical phenotype observed in the patients carrying these mutations can be explained by reduced binding efficacy of the transcription factors for the *F7* promoter[68].

By developing advanced *in silico* analysis methods and genotype-phenotype association studies, more comprehensive data on the effects of *F7* gene variants on the function of FVII protein are being provided[69,70]. Tiscia *et al*.[70] have described molecular consequences related to novel variants detected in FVII deficiency patients by using the bioinformatics software, including PROMO, SIFT, and PolyPhen-2. Structural characteristics of the mutant FVII proteins have also evaluated by *in silico* functional analysis on SPDB viewer software. The data of an *in silico* study predicted a possible damaging effect of the Cys400Ser missense mutation on the conformation of FVIIa via disrupting the Cys400-Cys428 disulfide bond. Very recently, the association of FVIIa levels with the incidence of coronary heart disease and the mortality rate of ischemic stroke has been assessed by Olson *et al*.[69]. They performed a genome-wide single nucleotide polymorphisms association analysis for FVIIa in European-Americans (n = 2410) patients and reported that rs1755685 in the *F7* promoter region on chromosome 13 was the most significantly relevant single nucleotide polymorphism to FVIIa levels. Interestingly, a functional *in vitro* site-directed mutagenesis study has previously demonstrated that allelic variants rs1755685 may increase *F7* gene expression[48]. Overall, various functional analysis methods may be implemented for the evaluation of each variant in *F7* gene. However, the best choice depends on the nature of the variant, the genotype-phenotype correlation in the patients, as well as previous studies on the population of interest.

Though the mutational spectrum of *F7* gene has been substantially described, the genotype-phenotype correlation in patients with FVII deficiency and the functional defects of the mutant FVII protein have yet to be precisely elucidated. This attempt may be more complicated in symptomatic patients with heterozygote variants. The pathogenicity and clinical severity of each particular *F7* gene variant should be evaluated considering overall data provided by *in vitro* and *in silico* functional analyses, as well as the presence of other interfering variants throughout the patients’ genome.

## References

[1] Getz TM, Piatt R, Petrich BG, Monroe D, Mackman N, Bergmeier W (2015). Novel mouse hemostasis model for real-time determination of bleeding time and hemostatic plug composition. Journal of thrombosis and haemostasis.

[2] Monie DD, DeLoughery EP (2017). Pathogenesis of thrombosis:cellular and pharmacogenetic contributions. Cardiovascular diagnosis and therapy.

[3] Mackman N, Tilley RE, Key NS (2007). Role of the extrinsic pathway of blood coagulation in hemostasis and thrombosis. Arteriosclerosis, thrombosis, and vascular biology.

[4] Ten Cate H, Hackeng TM, García de Frutos P (2017). Coagulation factor and protease pathways in thrombosis and cardiovascular disease. Thrombosis and haemostasis.

[5] Danziger J (2008). Vitamin K-dependent proteins, warfarin, and vascular calcification. Clinical journal of the American society of nephrology.

[6] de Moerloose P, Schved JF, Nugent D (2016). Rare coagulation disorders:fibrinogen, factor VII and factor XIII. Haemophilia.

[7] Franchini M, Marano G, Pupella S, Vaglio S, Masiello F, Veropalumbo E, Piccinini V, Pati I, Catalano L, Liumbruno GM (2018). Rare congenital bleeding disorders. Annals of translational medicine.

[8] Ustinov NB, Zav'yalova EG, Kopylov AM (2016). Effect of thrombin inhibitors on positive feedback in the coagulation cascade. Biochemistry (Mosco).

[9] Davie EW, Fujikawa K, Kisiel W (1991). The coagulation cascade:initiation, maintenance, and regulation. Biochemistry.

[10] Heinz S, Braspenning J (2015). Measurement of blood coagulation factor synthesis in cultures of human hepatocytes. Methods in molecular biology.

[11] Hatton MW, Blajchman MA, Sridhara S, Southward SM, Ross B, Kulzcycky M, Clarke BJ (2001). Metabolism of rabbit plasma-derived factor VII in relation to prothrombin in rabbits. American journal of physiology endocrinology and metabolism.

[12] Yang L, Li Y, Bhattacharya A, Zhang Y (2016). A plasma proteolysis pathway comprising blood coagulation proteases. Oncotarget.

[13] Kemball-Cook G, Johnson DJ, Tuddenham EG, Harlos K (1999). Crystal structure of active site-inhibited human coagulation factor VIIa (des-Gla). Journal of structural biology.

[14] Nemerson Y (1988). Tissue factor and hemostasis. Blood.

[15] Zelaya H, Rothmeier AS, Ruf W (2018). Tissue factor at the crossroad of coagulation and cell signaling. Journal of thrombosis and haemostasis.

[16] Bolt G, Steenstrup TD, Kristensen C (2007). All post-translational modifications except propeptide cleavage are required for optimal secretion of coagulation factor VII. Thrombosis and haemostasis.

[17] Kaufman RJ (1998). Post-translational modifications required for coagulation factor secretion and function. Thrombosis and haemostasis.

[18] D'Andrea G, Bossone A, Lupone MR, Peyvandi F, Maisto G, Perricone F, Grandone E, Margaglione M (2004). Molecular characterization of a factor VII deficient patient supports the importance of the second epidermal growth factor-like domain. Haematologica.

[19] Naderi A (2015). Coagulation factor VII is regulated by androgen receptor in breast cancer. Experimental cell research.

[20] Koizume S, Miyagi Y (2014). Breast cancer phenotypes regulated by tissue factor-factor VII pathway:possible therapeutic targets. World journal of clinical oncology.

[21] Eroğlu A, Oztürk A, Akar N (2011). Association between the -402GA, -401GT, and -323ins10-bp polymorphisms of factor VII gene and breast cancer. Breast cancer.

[22] Yokota N, Koizume S, Miyagi E, Hirahara F, Nakamura Y, Kikuchi K, Ruf W, Sakuma Y, Tsuchiya E, Miyagi Y (2009). Self-production of tissue factor-coagulation factor VII complex by ovarian cancer cells. British journal of cancer.

[23] Koizume S, Jin MS, Miyagi E, Hirahara F, Nakamura Y, Piao JH, Asai A, Yoshida A, Tsuchiya E, Ruf W, Miyagi Y (2006). Activation of cancer cell migration and invasion by ectopic synthesis of coagulation factor VII. Cancer research.

[24] John A, Gorzelanny C, Bauer AT, Schneider SW, Bolenz C (2017). Role of the coagulation system in genitourinary cancers:review. Clinical genitourinary cancer.

[25] Wu B, Zhou H, Hu L, Mu Y, Wu Y (2013). Involvement of PKCalpha activation in TF/VIIa/PAR2-induced proliferation, migration, and survival of colon cancer cell SW620. Tumour biology.

[26] Sevenet PO, Kaczor DA, Depasse F (2017). Factor VII deficiency:from basics to clinical laboratory diagnosis and patient management. Clinical and applied thrombosis/hemostasis.

[27] Traivaree C, Monsereenusorn C, Meekaewkunchorn A, Laoyookhong P, Suwansingh S, Boonyawat B (2017). Genotype and phenotype correlation in intracranial hemorrhage in neonatal factor VII deficiency among Thai children. The application of clinical genetics.

[28] Mariani G, Bernardi F (2009). Factor VII deficiency. Seminars in thrombosis and hemostasis.

[29] Tripathi P, Mishra P, Ranjan R, Tyagi S, Seth T, Saxena R (2019). Factor VII deficiency-an enigma;clinico-hematological profile in 12 cases. Hematology.

[30] Herrmann FH, Wulff K, Auerswald G, Schulman S, Astermark J, Batorova A, Kreuz W, Pollmann H, Ruiz-Saez A, De Bosch N, Salazar-Sanchez L, Greifswald Factor FVII Deficiency Study Group (2009). Factor VII deficiency:clinical manifestation of 717 subjects from Europe and Latin America with mutations in the factor 7 gene. Haemophilia.

[31] Kulkarni A, Lee CA, Griffeon A, Kadir RA (2006). Disorders of menstruation and their effect on the quality of life in women with congenital factor VII deficiency. Haemophilia.

[32] Mariani G, Dolce A, Marchetti G, Bernardi F (2004). Clinical picture and management of congenital factor VII deficiency. Haemophilia.

[33] Mariani G, Herrmann FH, Dolce A, Batorova A, Etro D, Peyvandi F, Wulff K, Schved JF, Auerswald G, Ingerslev J, Bernardi F, International Factor VII Deficiency Study Group (2005). Thrombosis and haemostasis.

[34] Naftalin J, Kadir R Life-threatening menorrhagia secondary to factor VII deficiency and leiomyomata. Journal of obstetrics and gynaecology 2006.

[35] Napolitano M, Di Minno MN, Batorova A, Dolce A, Giansily-Blaizot M, Ingerslev J (2016). Women with congenital factor VII deficiency:clinical phenotype and treatment options from two international studies. Haemophilia.

[36] Quintavalle G, Riccardi F, Rivolta GF, Martorana D, Di Perna C, Percesepe A (2017). F7 gene variants modulate protein levels in a large cohort of patients with factor VII deficiency. Results from a genotype-phenotype study. Thrombosis and haemostasis.

[37] Mariani G, Herrmann FH, Bernardi F, Schved JF, Auerswald G, Ingerslev J (2000). Clinical manifestations, management, and molecular genetics in congenital factor VII deficiency:the International Registry on Congenital Factor VII Deficiency (IRF7). Blood.

[38] Di Minno MND, Ambrosino P, Myasoedova V, Amato M, Ventre I, Tremoli E, Minno AD (2017). Recombinant activated factor VII (Eptacog Alfa Activated, NovoSeven®) in patients with rare congenital bleeding disorders. A systematic review on its use in surgical procedures. Current pharmaceutical design.

[39] Peyvandi F, Menegatti M (2016). Treatment of rare factor deficiencies in 2016. Hematology American society of hematology education program.

[40] Hagen FS, Gray CL, O'Hara P, Grant FJ, Saari GC, Woodbury RG, Hart CE, Insley M, Kisiel W, Kurachi K (1986). Characterization of a cDNA coding for human factor VII. Proceedings of the national academy of sciences of the United States of America.

[41] O'Hara PJ, Grant FJ, Haldeman BA, Gray CL, Insley MY, Hagen FS, Murray MJ (1987). Nucleotide sequence of the gene coding for human factor VII, a vitamin K-dependent protein participating in blood coagulation. Proceedings of the national academy of sciences of the United States of America.

[42] S S, R M, K K, A M (2017). Molecular characterization of Iranian patients with inherited coagulation factor VII deficiency. Balkan journal of medical genetics.

[43] Barbon E, Pignani S, Branchini A, Bernardi F, Pinotti M, Bovolenta M (2016). An engineered tale-transcription factor rescues transcription of factor VII impaired by promoter mutations and enhances its endogenous expression in hepatocytes. Scientific reports.

[44] Giansily-Blaizot M, Lopez E, Viart V, Chafa O, Tapon-Bretaudiere J, Claustres M, Taulan M (2012). Lethal factor VII deficiency due to novel mutations in the F7 promoter:functional analysis reveals disruption of HNF4 binding site. Thrombosis and haemostasis.

[45] Friso S, Lotto V, Choi SW, Girelli D, Pinotti M, Guarini P, Udali S, Pattini P, Pizzolo F, Martinelli N, Corrocher R, Bernardi F, Olivieri O (2012). Promoter methylation in coagulation F7 gene influences plasma FVII concentrations and relates to coronary artery disease. Journal of medical genetics.

[46] Eroğlu A, Oztürk A, Cam R, Akar N (2010). No significant association between the promoter region polymorphisms of factor VII gene and risk of venous thrombosis in cancer patients. Experimental oncology.

[47] Sabater-Lleal M, Chillón M, Howard TE, Gil E, Almasy L, Blangero J, Fontcuberta J, Soria JM (2007). Functional analysis of the genetic variability in the F7 gene promoter. Atherosclerosis.

[48] Bozzini C, Girelli D, Bernardi F, Ferraresi P, Olivieri O, Pinotti M, Martinelli N, Manzato F, Friso S, Villa G, Pizzolo F, Beltrame F, Corrocher R (2004). Influence of polymorphisms in the factor VII gene promoter on activated factor VII levels and on the risk of myocardial infarction in advanced coronary atherosclerosis. Thrombosis and haemostasis.

[49] Fagan K, Wilkinson I, Allen M, Brownlea S (1988). The coagulation factor VII regulator is located on 8p23.1. Human genetics.

[50] McVey JH, Boswell E, Mumford AD, Kemball-Cook G, Tuddenham EG (2001). Factor VII deficiency and the FVII mutation database. Human mutation.

[51] O'Brien DP, Gale KM, Anderson JS, McVey JH, Miller GJ, Meade TW, Tuddenham EG (1991). Purification and characterization of factor VII 304-Gln:a variant molecule with reduced activity isolated from a clinically unaffected male. Blood.

[52] Cramer TJ, Anderson K, Navaz K, Brown JM, Mosnier LO, von Drygalski A (2016). Heterozygous congenital Factor VII deficiency with the 9729del4 mutation, associated with severe spontaneous intracranial bleeding in an adolescent male. Blood cells, molecules and diseases.

[53] Peyvandi F, Jenkins PV, Mannucci PM, Billio A, Zeinali S, Perkins SJ, Perry DJ (2000). Molecular characterisation and three-dimensional structural analysis of mutations in 21 unrelated families with inherited factor VII deficiency. Thrombosis and haemostasis.

[54] Chollet ME, Andersen E, Skarpen E, Myklebust CF, Koehler C, Morth JP, Chuansumrit A, Pinotti M, Bernardi F, Thiede B, Sandset PM, Skretting G (2018). Factor VII deficiency:Unveiling the cellular and molecular mechanisms underlying three model alterations of the enzyme catalytic domain. Biochimica et biophysica acta Molecular basis of disease.

[55] Fromovich-Amit Y, Zivelin A, Rosenberg N, Tamary H, Landau M, Seligsohn U (2004). Characterization of mutations causing factor VII deficiency in 61 unrelated Israeli patients. Journal of thrombosis and haemostasis.

[56] O'Brien DP, Kemball-Cook G, Hutchinson AM, Martin DM, Johnson DJ, Byfield PG, Takamiya O, Tuddenham EG, McVey JH (1994). Surface plasmon resonance studies of the interaction between factor VII and tissue factor. Demonstration of defective tissue factor binding in a variant FVII molecule (FVII-R79Q). Biochemistry.

[57] Chaing S, Clarke B, Sridhara S, Chu K, Friedman P, VanDusen W, Roberts HR, Blajchman M, Monroe DM, High KA (1994). Severe factor VII deficiency caused by mutations abolishing the cleavage site for activation and altering binding to tissue factor. Blood.

[58] Kavlie A, Orning L, Grindflek A, Stormorken H, Prydz H (1998). Characterization of a factor VII molecule carrying a mutation in the second epidermal growth factor-like domain. Thrombosis and haemostasis.

[59] Peyvandi F, De Cristofaro R, Garagiola I, Palla R, Akhavan S, Landolfi R, Mannucci PM (2004). The P303T mutation in the human factor VII (FVII) gene alters the conformational state of the enzyme and causes a severe functional deficiency. British journal of haematology.

[60] Bharadwaj D, Iino M, Kontoyianni M, Smith KJ, Foster DC, Kisiel W (1996). Factor VII central. A novel mutation in the catalytic domain that reduces tissue factor binding, impairs activation by factor Xa, and abolishes amidolytic and coagulant activity. The Journal of biological chemistry.

[61] Mashayekhi A, Shahbazi S, Omrani M (2018). Functional and molecular characterization of C91S mutation in the second epidermal growth factor-like domain of factor VII. Iranian journal of biotechnology.

[62] Millar DS, Kemball-Cook G, McVey JH, Tuddenham EG, Mumford AD, Attock GB, Reverter JC, Lanir N, Parapia LA, Reynaud J, Meili E, von Felton A, Martinowitz U, Prangnell DR, Krawczak M, Cooper DN (2000). Molecular analysis of the genotype-phenotype relationship in factor VII deficiency. Human genetics.

[63] Chang JY, Stafford DW, Straight DL (1995). The roles of factor VII's structural domains in tissue factor binding. Biochemistry.

[64] Wu Y, Tu X, Lian Y, Chen F, Lan F, Zhu Z (2006). Characterization of a Cys329Gly mutation causing hereditary factor VII deficiency. Acta haematologica.

[65] Au WY, Lam CC, Chan EC, Kwong YL (2000). Two novel factor VII gene mutations in a Chinese family with factor VII deficiency. British journal of haematology.

[66] Mashayekhi A, Shahbazi S, Omrani M, Mahdian R (2018). *In vitro* expression of mutant factor VII proteins and characterization of their clinical significance. Molecular medicine reports.

[67] Carew JA, Pollak ES, Lopaciuk S, Bauer KA (2000). A new mutation in the HNF4 binding region of the factor VII promoter in a patient with severe factor VII deficiency. Blood.

[68] Olson NC, Raffield LM, Lange LA, Lange EM, Longstreth WT, Chauhan G, Debette S, Seshadri S, Reiner AP, Tracy RP (2018). Associations of activated coagulation factor VII and factor VIIa-antithrombin levels with genome-wide polymorphisms and cardiovascular disease risk. Journal of thrombosis and haemostasis.

[69] Tiscia G, Favuzzi G, Chinni E, Colaizzo D, Fischetti L, Intrieri M, Margaglione M, Grandone E (2017). Factor VII deficiency:a novel missense variant and genotype-phenotype correlation in patients from Southern Italy. Human genome variation.

[70] Perry DJ (2002). Factor VII deficiency. British journal of haematology.

[71] Cavallari N, Balestra D, Branchini A, Maestri I, Chuamsunrit A, Sasanakul W, Mariani G, Pagani F, Bernardi F, Pinotti M (2012). Activation of a cryptic splice site in a potentially lethal coagulation defect accounts for a functional protein variant. Biochimica et biophysica acta.

[72] Peyvandi F, Garagiola I, Palla R, Marziliano N, Mannucci PM (2005). Role of the 2 adenine (g.11293_11294insAA) insertion polymorphism in the 3'untranslated region of the factor VII (FVII) gene:molecular characterization of a patient with severe FVII deficiency. Human mutation.

[73] Giansily-Blaizot M, Aguilar-Martinez P, Briquel ME, d'Oiron R, De Maistre E, Epelbaum S, Schved JF (2003). Two novel cases of cerebral haemorrhages at the neonatal period associated with inherited factor VII deficiency, one of them revealing a new nonsense mutation (Ser52Stop). Blood coagulation and fibrinolysis.

[74] Carew JA, Pollak ES, High KA, Bauer KA (1998). Severe factor VII deficiency due to a mutation disrupting an Sp1 binding site in the factor VII promoter. Blood.

